# Predictive factors of severe coronavirus disease 2019 in previously healthy young adults: a single-center, retrospective study

**DOI:** 10.1186/s12931-020-01412-1

**Published:** 2020-06-22

**Authors:** Changzhi Zhou, Zhe Huang, Weijun Tan, Xueying Li, Wen Yin, Yang Xiao, Zhaowu Tao, Shuang Geng, Yi Hu

**Affiliations:** 1 Department of Respiratory and Critical Care Medicine, The Central Hospital of Wuhan, Tongji Medical College, Huazhong University of Science and Technology, Shengli Street No. 26, Jiang’an District, Wuhan, 430014 Hubei Province China; 2 Cardiac Function Department, The Central Hospital of Wuhan, Tongji Medical College, Huazhong University of Science and Technology, Shengli Street No. 26, Jiang’an District, Wuhan, 430014 Hubei Province China

**Keywords:** COVID-19, SARS-CoV-2, Predictive factors, Retrospective study

## Abstract

**Background:**

Several previously healthy young adults have developed Coronavirus Disease 2019 (COVID-19), and a few of them progressed to the severe stage. However, the factors are not yet determined.

**Method:**

We retrospectively analyzed 123 previously healthy young adults diagnosed with COVID-19 from January to March 2020 in a tertiary hospital in Wuhan. Patients were classified as having mild or severe COVID-19 based on their respiratory rate, SpO_2_, and PaO_2_/FiO_2_ levels. Patients’ symptoms, computer tomography (CT) images, preadmission drugs received, and the serum biochemical examination on admission were compared between the mild and severe groups. Significant variables were enrolled into logistic regression model to predict the factors affecting disease severity. A receiver operating characteristic (ROC) curve was applied to validate the predictive value of predictors.

**Result:**

Age; temperature; anorexia; and white blood cell count, neutrophil percentage, platelet count, lymphocyte count, C-reactive protein, aspartate transaminase, creatine kinase, albumin, and fibrinogen values were significantly different between patients with mild and severe COVID-19 (*P* < 0.05). Logistic regression analysis confirmed that lymphopenia (*P* = 0.010) indicated severe prognosis in previously healthy young adults with COVID-19, with the area under the curve (AUC) was 0.791(95% Confidence Interval (CI) 0.704–0.877)(*P* < 0.001).

**Conclusion:**

For previously healthy young adults with COVID-19, lymphopenia on admission can predict severe prognosis.

## Background

From December 2019 to March 2020, coronavirus disease 2019 (COVID-19) has been considered an epidemic in China, specifically in Wuhan City, Hubei Province, where this disease initially emerged [1]. According to data, this disease has already been considered a global epidemic because more than 200 countries have been affected, with more than 970,000 patients infected and 50,000+ deaths [2]. Hence, the severe acute respiratory syndrome coronavirus-2 (SARS-CoV-2) which caused COVID-19, shows stronger infectivity than SARS-CoV and Middle East respiratory syndrome (MERS)-CoV, although, the mortality rate of COVID-19 is lower than SARS and MERS [3]. Nevertheless, COVID-19 should be given careful attention considering that its mortality rate is increasing [4]. The previous study has reported that elderly men, specifically those with chronic comorbidities, tend to develop a more severe COVID-19 which may be fatal compared with young men [5], considering that these elderly men have impaired immune response and incomplete functional organs. However, several previously healthy young adults who developed severe COVID-19, required superior oxygen therapy, including high-flow nasal cannula (HFNC), noninvasive positive-pressure ventilation (NPPV), invasive positive-pressure ventilation (IPPV), and even extracorporeal membrane oxygenation (ECMO). The reason this happened remains unclear. Thus, we used our hospital’s data to determine the factors affecting patients’ clinical outcomes using an appropriate statistical model.

## Patients and methods

### Study design

**Fig. 1:**
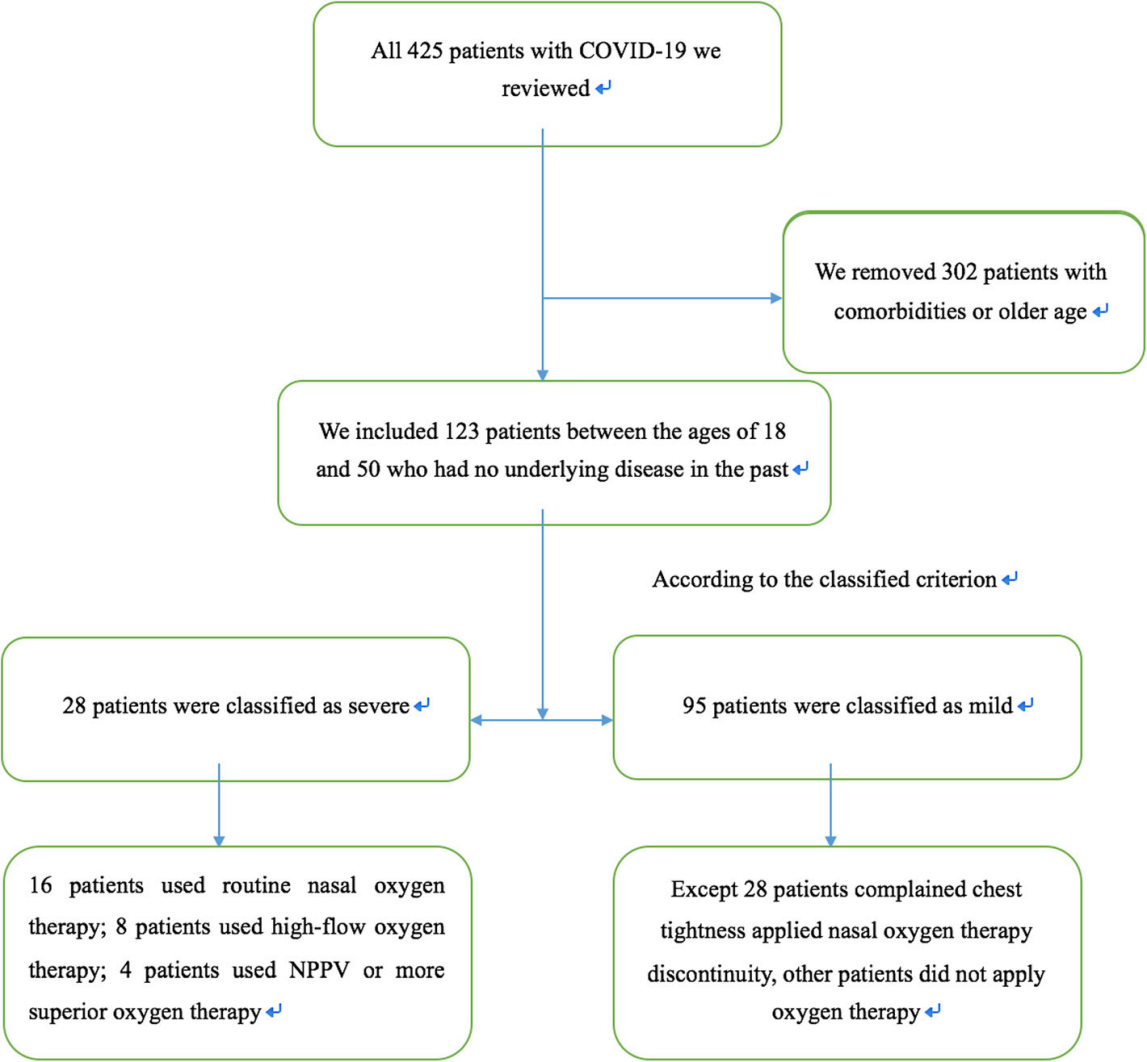
Recruitment flowchart of patients for the study

We conducted a retrospective study in a single-center, the Central Hospital of Wuhan. Moreover, this hospital was one of the earliest tertiary hospitals that admitted COVID-19 patients in Wuhan in December 2019. A total of 425 patients were included in this study from January 1, 2020 to March 28, 2020, of whom 123 patients aged 18 to 50 years were considered previously healthy adults. (Fig. 1) Our exclusion criteria included chronic diseases: hypertension, diabetes, coronary heart disease, chronic cerebrovascular disease, chronic respiratory disease (asthma, COPD), chronic hepatitis, and other chronic diseases that can affect the immune status. They were diagnosed with COVID-19 based on the guidelines of the World Health Organization (WHO) [6]. We extracted the medical records and charts of each patient; a team of physicians who had been treating COVID-19 patients reviewed all the data. Further, because the study is a retrospective study and does not involve patients’ privacy, the informed consent was waived.

### Clinical and biological data

Patients’ medical history was retrospectively reviewed (mainly by ZCZ and HZ), and their demographic information was collected. Symptoms such as fever (the highest temperature recorded), cough, expectoration, headache, wheezing, weakness, muscle ache, pharyngalgia, runny nose, anorexia, stethalgia, chest tightness, dyspnea, and diarrhea were assessed. Time from illness onset to hospital admission (days), computed tomography (CT) scan revealing bilateral lesion or unilateral lesion, and preadmission drugs used were also evaluated. While receiving treatment, all patients underwent laboratory testing on admission day or the next morning. Throat swab specimens were routinely collected, tested by real-time polymerase chain reaction for SARS-CoV-2 RNA detection within 12 h on admission, and other examination data were assessed in 24 h on admission. Moreover, serum biochemistry analysis was performed. WBC count, N%, and N, lymphocyte, and PLT were assessed on formal full blood examination. Levels of creatinine (Cr), blood urea nitrogen (BUN), total bilirubin (TBIL), direct bilirubin (DBIL), indirect bilirubin (IBIL), albumin (ALB), aspartate transaminase (AST), alanine transaminase (ALT), creatine kinase (CK), creatine kinase isoenzyme (CK-MB), D-dimer, and fibrinogen (Fib), prothrombin time (PT), and international normalized ratio (INR) on admission were also recorded. All the data were checked by another researcher to ascertain their accuracy.

In 123 selected patients, the earliest and latest admission dates were January 1, 2020 and March 17, 2020, respectively. Moreover, these patients were followed up until March 28, 2020. Our observed endpoint event was the patients being diagnosed with severe COVID-19 durning the whole inpatient period according to the clinical criterion. Any missing data were recorded as unknown.

Patients were diagnosed with severe COVID-19 if they presented with (a) dyspnea with respiratory rate ≥ 30 breaths/min, (b) finger oxygen saturation ≤ 93% at resting state, and (c) arterial partial pressure of oxygen/fractional concentration of inspired oxygen ≤300 mmHg (1 mmHg = 0.133 kPa) [7].

### Statistical analyses

All analyses were performed using the Statistical Package for the Social Sciences Statistic (SPSS) version 24.0 (Chicago, IL). Continuous data were illustrated as means and standard deviations (mean, standard deviation [SD]). Categorical data were illustrated as counts and percentages. Descriptive statistics were illustrated as mean (standard deviation, SD) or median (interquartile range) according to data distribution. Hypothesis testing for patient-level data was performed using the chi-squared test for categorical variables, Student’s t-test for normally distributed data, and Wilcoxon rank-sum test for non-normally distributed data. Characteristics of mild and severe COVID-19 patients were compared, and we used a logistic regression model to determine the strongest predictive factors of the severity of the disease. A receiver operating characteristic (ROC) curve was used to validate the predictive value of predictors. All reported *P* values were two-tailed.

## Results

### Demographic characteristics

Based on the data we collected, a total of 28 out of the 123 (22.76%) previously healthy young patients developed severe COVID-19. Of those 28, 20 patients of severe disease were diagnosed with the PaO2/FiO2 ≤ 300 mmHg, four patients were classified with rapid breath rate ≥ 30 breaths/min, and four with SpO2 ≤ 93% in resting time. All patients received appropriate treatment, and severe disease patients received oxygen therapy. Sixteen patients received routine nasal oxygen, and eight patients needed high-flow oxygen therapy to alleviate their symptoms or hypoxia. However, the other four patients needed NPPV or superior oxygen therapy. (Fig. 1) The mean age of all patients was approximately 36.61 (range, 22–50) years, and 55 of the 123 (44.7%) patients were male. All patients were residents of Wuhan City. Fever was the most common symptom (79.7%), followed by cough (60.9%), anorexia (48%), and weakness (40.7%). Pharyngalgia (9.8%), stethalgia (8.1%), dyspnea (5.7%), and runny nose (1.6%) were rarely observed in previously healthy young patients during hospital admission.

The symptoms observed between the two groups were compared. Severe patients were observed to experience fever more (96.2% vs. 73.8%, *P* = 0.014) and anorexia (76.9% vs. 36.9%, *P* < 0.001), had higher temperature (38.5[0.5] vs. 38.1[0.8], *P* = 0.036), and were older (39.5[5.35] vs. 35[6], *P* = 0.019) compared with mild patients. Most CT photographs showed a few ground-glass opacities (GGO) bilateral or unilateral. We compared the bilateral lesion of the mild group 63/95 (66.3%) to with that of the severe group 21/28 (75%) and detected no difference (*P* > 0.05). (Table 1).

**Table 1:** Difference between mild and severe patients based on initial symptom and serum biochemical examination

Variables	All (*n* = 123)	Mild (*n* = 95)	Severe (*n* = 28)	*P*
**Characteristic**				
Age (years)	37 (7)	35 (6)	39.5 (5.35)	0.019
Sex (male)	55/123 (44.7%)	38/95 (40%)	17/28 (60.7%)	0.053
**Signs and symptoms**				
Temp(°C)	38.20 (0.80)	38.10 (0.8)	38.50 (0.50)	0.036
Time pre-adm (days)	6 (4)	7 (4)	6.00 (2.50)	0.297
Fever	98/123 (79.7%)	71/95 (74.7%)	27/28 (96.4%)	0.012
Cough	79/123 (64.2%)	64/95 (67.4%)	15/28 (53.6%)	0.181
Expectoration	32/123 (26%)	23/95 (24.2%)	9/28 (32.1%)	0.400
Headache	19/123 (15.4%)	15/95 (15.8%	4/28 (14.3%)	0.847
Wheeze	24/123 (19.5%)	18/95 (18.9%)	6/28 (21.4%)	0.771
Weakness	50/123 (40.7%)	36/95 (37.9%)	14/28 (50%)	0.252
Muscle ache	31/123 (25.2%)	25/95 (26.3%)	6/28 (21.4%)	0.601
Pharyngalgia	12/123 (9.8%)	9/95 (9.5%)	3/28 (10.7%)	0.846
Runny nose	2/123 (1.6%)	2/95 (2.1%)	0/28 (0%)	0.439
Anorexia	59/123 (48.0%)	38/95 (40%)	21/28 (75%)	0.001
Stethalgia	10/123 (8.1%)	9/95 (9.5%)	1/28 (3.6%)	0.315
Chest Tightness	39/123 (31.7%)	28/95 (29.5%)	11/28 (39.3%)	0.327
Dyspnea	7/123 (5.7%)	5/95 (5.3%)	2/28 (7.1%)	0.706
Diarrhea	13/123 (10.6%)	9/95 (9.5%)	4/28 (14.3%)	0.467
CT Bilateral Lesion	84/123 (68.3%)	63/95 (66.3%)	21/28 (75%)	0.385
Preadmission Drugs	49/123 (39.8%)	40/95 (42.1%)	9/28 (32.1%)	0.344
**Serum Biomarkers**				
WBC(10^9^/L)	4.85 (1.04)	5.01 (0.93)	3.97 (1.37)	0.048
RBC(10^9^/L)	4.49 (0.40)	4.47 (0.39)	4.69 (0.45)	0.102
N%	63.77 (15.16)	61.99 (13.93)	69.81 (17.72)	0.016
N(10^9^/L)	2.96 (0.89)	3.01 (0.77)	2.57 (1.81)	0.563
L(10^9^/L)	1.22 (0.41)	1.30 (0.56)	0.80 (0.36)	< 0.001
PLT(10^9^/L)	173 (39)	184 (42)	157.50 (23.00)	0.016
CRP (mg/dL)	0.58 (1.66)	0.46 (0.93)	1.46 (2.58)	< 0.001
Cr (umol/L)	64.50 (8.30)	63.30 (9.20)	66.35 (10.63)	0.433
BUN (mmol/L)	3.62 (0.58)	3.62 (0.56)	3.83 (1.23)	0.173
AST(U/L)	20.00 (6.20)	19.00 (6.30)	22.45 (11.83)	0.037
ALT(U/L)	18.50 (11.20)	17.70 (12.00)	24.05 (7.78)	0.094
TBIL (umol/L)	8.80 (3.40)	8.80 (2.60)	8.65 (3.80)	0.686
DBIL (umol/L)	3.00 (0.90)	3.00 (0.80)	3.05 (1.43)	0.319
IBIL (umol/L)	5.60 (2.60)	5.60 (2.60)	5.75 (3.43)	0.959
ALB(g/L)	41.96 (4.62)	42.42 (4.48)	40.40 (4.82)	0.042
CK(U/L)	67 (51)	64.50 (45.75)	92.00 (106.00)	0.011
CK-MB(U/L)	7.00 (3.00)	6.90 (3.70)	8.00 (2.00)	0.243
D-dimer (mg/dL)	0.32 (0.28)	0.30 (0.46)	0.37 (0.15)	0.574
PT(s)	16.20 (0.60)	16.20 (0.60)	15.8 (1.20)	0.330
INR	0.98 (0.07)	0.99 (0.07)	0.98 (0.07)	0.414
Fib(g/L)	2.52 (0.42)	2.50 (0.30)	2.75 (0.76)	0.001

All data are expressed as n (%), median (interquartile range), and mean (standard deviation). The missing date: CRP, PT, INR and Fib (mild 1case, severe 1case), CK and CK-MB (mild 3 cases, severe 1case), D-dimer (mild 0 cases, severe 1case). Where N is the total number of patients with available data. *P* values comparing mild and severe are from χ2 or Mann-Whitney U test. *ALB* albumin, *ALT* alanine transaminase, *AST* aspartate transaminase, *BUN* blood urea nitrogen, *CK* creatine kinase, *CK-MB* creatine kinase isoenzyme, *Cr* creatinine, *CRP* C-reactive protein, *DBIL* direct bilirubin, *Fib* fibrinogen, *IBIL*, indirect bilirubin, *INR* international normalized ratio, *L* lymphocyte count, *N* neutrophil count, *N%* neutrophil%, *PLT* platelet, *PT* prothrombin time, *RBC* red blood cell, *TBIL* total bilirubin, *Temp* temperature, *WBC* white blood cell

### Biochemical examination

Regarding the serum biomarkers, WBC (5.01 [0.93] vs. 3.97 [1.37], *P* = 0.048) (Normal range 3.5–9.5*10^9^/L), N% (61.99[13.93] vs. 69.81[17.72], *P* = 0.016) (Normal range 40–75%), lymphocyte count (1.30[0.56] vs. 0.80[0.36], *P* < 0.001) (Normal range 1.1–3.2*10^9^/L), and levels of PLT count (184[42] vs. 157.50[23], *P* = 0.016) (Normal range 125–350*10^9^/L), C-reactive protein (CRP) (0.46[0.093] vs. 1.46[2.58], *P* < 0.001) (Normal range 0–0.6 mg/dL), AST (19[6.3] vs. 22.45[11.83], *P* = 0.037) (Normal range 9–50 U/L), ALB (42.42[4.48] vs. 40.40[4.82], *P* = 0.042) (Normal range 40–55 g/L), CK (64.50[45.75] vs. 92[106], *P* = 0.011) (Normal range 38–174 U/L), and Fib (2.50[0.3] vs. 2.75[0.76], *P* = 0.001) (Normal range 2–4 g/L) were different between mild and severe groups, respectively. All compared variables except lymphocyte count and CRP were within the normal range, therefor we considered CRP and lymphocyte count more meaningful. Among all variables, only the N% and ALB levels were normally distributed. (Table 1).

### Predictive factors affect severe prognosis

**Fig. 2:**
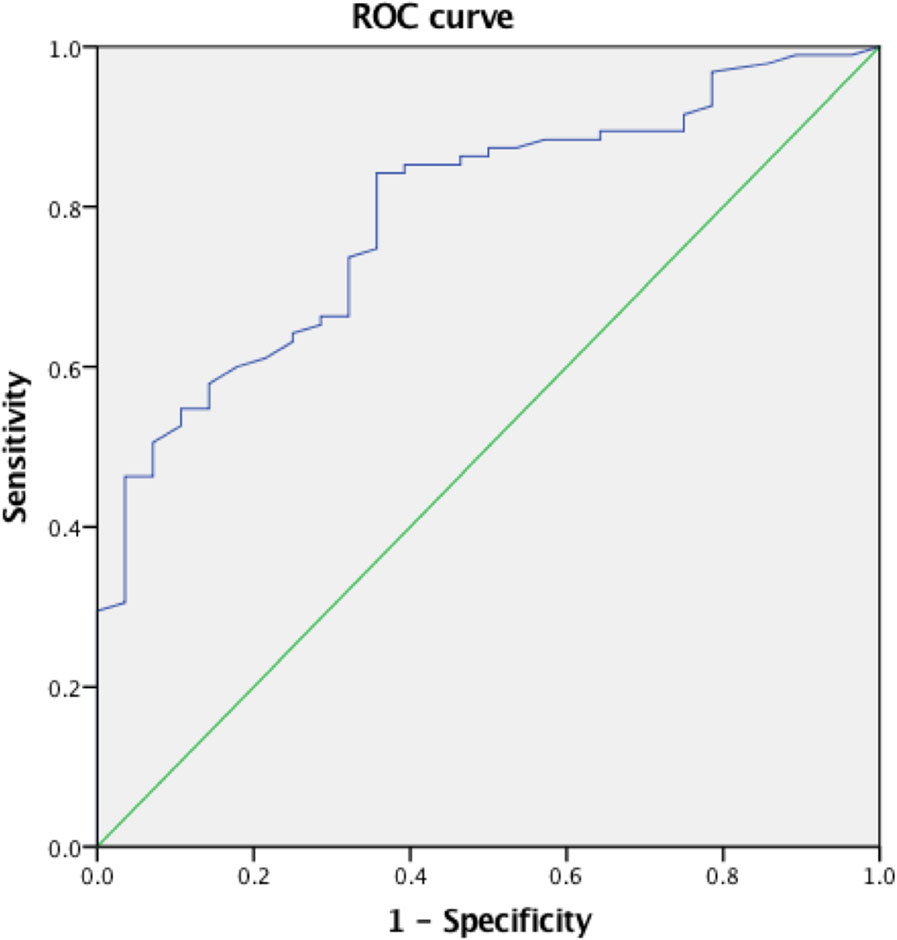
Lymphocyte count for severe progressed coronavirus disease in previously healthy adults using receiver operating characteristic curves. The area under the curve of lymphocyte count is 0.791(95% confidence interval: 0.704–0.877) (*P* < 0.001). The best cutoff for lymphocyte count for prediction 0.905*10^9^/L is with a specificity of 64.3% and a sensitivity of 84.2% (*P* < 0.001)

We subsequently enrolled all variables into a one-factor logistic regression to determine the significant variables. According to our results, age (odds ratio [OR], 1.066; 95% confidence interval [CI], 1.007–1.119; *P* = 0.027), temperature (OR, 1.685; 95% CI, 1.028–2.763; *P* = 0.038), N% (OR, 1.038; 95% CI, 1.007–1.071; *P* = 0.017), L (OR, 0.084; 95% CI, 0.025–0.280; *P* < 0.001), PLT (OR, 0.991; 95% CI, 0.983–0.999; *P* = 0.029), CRP (OR, 1.199; 95% CI, 1.046–1.375; *P* = 0.009), ALB (OR, 0.905; 95% CI, 0.820–0.998; *P* = 0.045), Fib (OR, 2.832; 95% CI, 1.438–5.578; *P* = 0.003), fever (OR, 9.127; 95% CI, 1.176–70.816; *P* = 0.034), and anorexia (OR, 4.5; 95% CI, 1.742–11.622; *P* = 0.002) individually contributed to the final severe outcome. (Table 2) Finally, a logistic regression analysis was performed again using a multifactor model that enrolled all significant variables in one-factor logistic regression to confirm lymphopenia, which was the strongest predictor of severe prognosis (OR, 0.084; 95% CI, 0.013–0.559; *P* = 0.010). (Table 3) The ROC curve was used to analyze the predictive value of lymphocyte count for determining severe COVID-19. The area under the ROC curve (AUC) was estimated, the result showed that the AUC was 0.791 (95% CI: 0.704–0.877), with a specificity of 64.3% and a sensitivity of 84.2%(*P* < 0.001) (Fig. 2). The cutoff value of the lymphocyte count was 0.905*10^9^/L.

**Table 2:** One-factor logistic regression enrolling all recorded variables

Variables	OR	95% CI		*P*
**Characteristic**				
Age	1.066	1.007	1.129	0.027
Sex (male)	2.318	0.979	5.491	0.056
**Sign and Symptom**				
Temp	1.685	1.028	2.763	0.038
Time pre-adm	0.955	0.880	1.035	0.260
Fever	9.127	1.176	70.816	0.034
Cough	0.559	0.237	1.318	0.184
Expectoration	1.483	0.590	3.727	0.402
Headache	0.889	0.269	2.932	0.847
Wheeze	1.167	0.413	3.295	0.771
Weakness	1.639	0.701	3.830	0.254
Muscle ache	0.764	0.278	2.100	0.601
Pharyngalgia	1.147	0.288	4.560	0.846
Runny nose	0.000	0.000	–	0.999
Anorexia	4.500	1.742	11.622	0.002
Stethalgia	0.354	0.043	2.921	0.335
Chest tightness	1.548	0.644	3.723	0.329
Dyspnea	1.385	0.254	7.556	0.707
Diarrhea	1.593	0.451	5.624	0.470
CT Bilateral Lesion	1.524	0.586	3.961	0.388
Preadmission drugs	0.651	0.267	1.589	0.346
**Serum Biomarkers**				
WBC	0.829	0.653	1.052	0.123
RBC	1.661	0.750	3.681	0.211
N%	1.038	1.007	1.071	0.017
N	1.006	0.810	1.249	0.956
L	0.084	0.025	0.280	< 0.001
PLT	0.991	0.983	0.999	0.029
CRP	1.199	1.046	1.375	0.009
Cr	1.003	0.997	1.010	0.320
BUN	1.020	0.954	1.092	0.560
AST	1.005	0.989	1.021	0.557
ALT	0.999	0.989	1.008	0.773
TBIL	1.027	0.940	1.123	0.548
DBIL	1.168	0.921	1.480	0.200
IBIL	1.007	0.889	1.140	0.910
ALB	0.905	0.820	0.998	0.045
CK	1.002	1.000	1.004	0.121
CK-MB	1.010	0.928	1.101	0.811
D-dimer	0.748	0.419	1.336	0.327
PT	0.871	0.700	1.085	0.219
INR	0.468	0.031	7.142	0.585
Fib	2.832	1.438	5.578	0.003

*OR* odds ratio, *CI* confidence interval, *ALB* albumin, *ALT* alanine transaminase, *AST* aspartate transaminase, *BUN* blood urea nitrogen, *CK* creatine kinase, *CK-MB* creatine kinase isoenzyme, *Cr* creatinine, *CRP* C-reactive protein, *DBIL* direct bilirubin, *Fib* fibrinogen, *IBIL* indirect bilirubin, *INR* international normalized ratio, *L* lymphocyte count, *N* neutrophil count, *N%* neutrophil%, *PLT* platelet, *PT* prothrombin time, *RBC* red blood cell, *TBIL* total bilirubin, *Temp* temperature, *WBC* white blood cell

**Table 3:** Multifactor logistic regression enrolling the significant variables in one-factor logistic regression

Variables	OR	95% CI		*P*
Age	1.065	0.986	1.150	0.108
Fever	2.472	0.136	44.840	0.540
Temp	0.724	0.323	1.622	0.432
Anorexia	1.603	0.471	5.456	0.450
L	0.084	0.013	0.559	0.010
PLT	0.992	0.981	1.003	0.157
CRP	0.936	0.746	1.175	0.569
N%	0.989	0.945	1.035	0.639
ALB	0.935	0.823	1.061	0.297
Fib	2.754	0.878	8.637	0.082

*OR* odds ratio, *CI* confidence interval, *ALB* albumin, *CRP* C-reactive protein, *Fib* fibrinogen, *L* lymphocyte count, *N%* neutrophil%, *PLT* platelet, *Temp* temperature

## Discussion

Coronaviruses are known because of the previously encountered SARS-CoV and MERS-CoV epidemics, and both are zoonotic viruses [8]. Similar to the previous two coronavirus outbreaks, fever and cough were the most common symptoms with viral pneumonia [9]. Our study showed that patients with severe COVID-19 had higher febrile temperatures with a large number of them in the overall population. For those previously healthy young adults with sound immunity, the occurrence of high fever after viral infection indicates the body’s rapid response against the invaded pathogen. It revealed that a fierce inflammatory reaction in patients was one of the factors leading to the “severe” status. Anorexia also showed significance in our study; we presumed that it co-occurred as a symptom with fever. Patients with high fever accompanied by anorexia could be discriminated more strongly from those with ordinary fever (*P* = 0.001). The previous study showed that dyspnea and chest tightness were indicators of severe COVID-19 [10]. Inversely, we found that both symptoms were insignificant to distinguish severe COVID-19 in previously healthy young adults. In fact, only seven patients (two in the severe group and five in the mild group) had dyspnea and 39 patients had chest tightness (11 in the severe group and 28 in the mild group) on admission. Owing to the relatively good state of the lungs, we inferred that there was less probability of respiratory decompensation on admission. Besides, a great sample size was needed for a more powerful prove. Furthermore, a larger cohort would be required to validate these associations. Additionally, we found that older patients would need to beware of aggravation of the disease because an older age indicates the decline in organ function as well as ability of the body to self-regulate.

Our study showed that the estimated rate of COVID-19 severity in patients was 22.76%, which was lower than that of the previous study [11]. This is possibly attributed to the single-center design of this study, and we collected the previously healthy young part of patients, causing possible bias in patient distribution. Moreover, based on the results of our study, the sex difference was not associated with the development of severe COVID-19, a result consistent with the previous study [11].

It is believed that previously healthy young adults usually have a sound immune system; thus, they can immediately and accurately respond to invading pathogens and viruses. However, the reasons they quickly develop respiratory failure or acute respiratory distress syndrome (ARDS) after being infected with SARS-Co-2 are still unclear. We presume that the first reason is possibly attributed to the pathophysiology of the viral load. Tiny viral loads allow the immune system to produce antibodies whether any clinical symptoms are experienced by the body or not [12]. However, when a significant number of viruses invade the body in a short time, the immune system will be overwhelmed, resulting in massive cytokine reaction that ultimately damages the lung’s tiny vessels. It will subsequently result in pulmonary edema, providing significant burden to the circulatory system, eventually crushing the heart and lungs as well as causing coagulation and massive tiny thromboses in the tiny vessels of the whole body. Recently, Zou et al. have proven the presence of viral load of the upper respiratory tract that was detected in the asymptomatic patient was similar to that in the symptomatic patients [13]. According to another previous study, the lower respiratory tract specimens usually have significantly higher viral loads and genome fractions than the upper respiratory tract specimens [14]. The second reason is possibly attributed to the different inflammatory responses of each individual, which also play a crucial role in coronavirus-induced lung injury and ARDS. CRP is a nonspecific marker of inflammation which was widely used as a biochemical indicator for reflecting the acute severe systemic inflammatory response caused by a viral infection, such that our research illustrated that the severe COVID-19 patients had a high value than mild ones. In 2003, corticosteroid was widely administered in the treatment of SARS to control pulmonary inflammatory edema by regulating the immunity responses toward SARS-CoV. Russell et al. announced that corticosteroids should be administered before an inflammatory storm occurs to prevent lung injury [15]. However, recently, most studies have reported that corticosteroids could only delay viral clearance [16] and are insignificantly associated with the mortality rate in severe viral pneumonia [17]. According to Wang et al.’s study [18] in comprising 46 COVID-19 patients, low-dose and short-term administration of corticosteroids was associated with a faster improvement of clinical symptoms and absorption of lung focus. However, patients may significantly benefit when the medication is administered at the right time with a reasonable dose.

Elevated D-dimer levels in COVID-19 patients, associated with poor clinical outcomes has been proven [19]. Tiny thromboses are produced by inflammatory cascade, blocking the pulmonary vessel, which might result in disseminated intravascular coagulation (DIC) without stopping inflammation. In fact, in clinical practice, low-molecular-weight heparin (LMWH) is administered to prevent thrombosis if the D-dimer levels at > 4 μg/ml. The inflammatory reaction includes a cytokine storm, resulting in internal environmental disruption and inducing coagulation maladjustment. Patients in the intensive care unit (ICU) or those who died may present a final phase of body decompensation, with elevated D-dimer. The study we conducted could be an early phase before coagulation decompensation. The increase in Fib levels and decrease in PLT counts could be a coagulation compensation before D-dimer elevating.

Lymphopenia is commonly assessed in most viral infections, specifically, type A and B influenza [20]. According to a previous study, lymphopenia was also observed in SARS and MERS [21]. Coronavirus infection usually induces an immune response, resulting in decreased CD4 count and immunosuppression [22, 23]. Simultaneously, the virus also damages the epithelial walls, and disrupts surfactant in the airways, providing access to rapid bacterial growth and resulting in a secondary bacterial infection, adversely affecting immunosuppressed patients.

Our study also has limitations. Firstly, considering this was a single-center, retrospective study with limited sample size, avoiding bias regarding patient distribution is considered difficult. Secondly, lymphopenia was observed in contribution to severe prognosis in this study, but data regarding CD4 and CD8 counts and other inflammatory biomarkers were not assessed; these biomarkers may also possibly associate with the patients’severe prognosis .

In summary, this is the first study to systematically describe the clinical symptoms and laboratory biomarkers of COVID-19 in mild and severe groups of previously healthy young adults. If the patients who were admitted to the hospital had higher fever and symptoms of anorexia, biochemical examination showed higher CRP and lymphopenia; the patients is then more likely to progress to severe COVID-19. Furthermore, lymphopenia was considered as the strongest predictor of severe prognosis. Our study findings are possibly beneficial for physicians to comprehensively understand the predictive factors associated with disease severity for COVID-19, allowing them to immediately and accurately provide supportive treatment, preventing the rapid development of the disease and decreasing the mortality rate. However, additional multicenter, prospective studies are required to further assess the clinical outcomes of severe COVID-19.

## Conclusion

In conclusion, lymphopenia is considered the strongest predictor of severe prognosis in previously healthy young adults diagnosed with COVID-19. For them, proper supervision and supportive treatment combined with superior oxygen therapy are required.

## Data Availability

The datasets analyzed during the current study were made available from the corresponding author on reasonable request.

## Abbreviations

ALB: Albumin
ALT: Alanine transaminase
AST: Aspartate transaminase
ARDS: Acute Respiratory Distress Syndrome
AUC: Area under the receiver operating characteristic curve
BUN: Blood urea nitrogen
CI: Confidence interval
CK: Creatine kinase
CK-MB: Creatine kinase isoenzyme
Cr: Creatinine
CRP: C-reactive protein
CT: Computed tomography
COVID-19: Coronavirus Disease 2019
DBIL: Direct bilirubin
ECMO: Extracorporeal membrane oxygenation
Fib: Fibrinogen
GGO: Ground-glass opacity
HFNC: High-flow nasal cannula
IBIL: Indirect bilirubin
DIC: Disseminated intravascular coagulation
ICU: Intensive care unit
INR: International normalized ratio
IPPV: invasive positive-pressure ventilation
IQR: Interquartile ranges
L: Lymphocyte count
LMWH: Low-molecular-weight heparin
MERS-COV: Middle East respiratory syndrome coronavirus
N: Neutrophil count
N%: Neutrophil%
NPPV: Noninvasive positive-pressure ventilation
OR: Odds ratio
PLT: Platelet
PT: Prothrombin time
RBC: Red blood cell
ROC: Receiver operating characteristic
SARS-CoV: Severe acute respiratory syndrome coronavirus
SARS-CoV-2: Severe acute respiratory syndrome coronavirus 2
SPSS: Statistical Package for the Social Sciences Statistic
TBIL: Total bilirubin
Temp: Temperature
WBC: White blood cell
WHO: World Health Organization
